# Perceived School Safety and Creative Thinking: The Sequential Mediating Roles of Cooperation and Curiosity in Finland and Hong Kong (China)

**DOI:** 10.3390/bs16081387

**Published:** 2026-08-12

**Authors:** Xinxiu Jiang, Xiaorui Huang

**Affiliations:** 1 College of Education for the Future, Beijing Normal University, Zhuhai 519087, China; 2 College of Education Science, Taizhou University, Taizhou 225300, China; 3 Collaborative Innovation Center of Assessment Toward Basic Education Quality, Beijing Normal University, Zhuhai 519087, China

**Keywords:** perceived school safety, school climate, creative thinking, cooperation, curiosity, multilevel serial mediation, PISA 2022

## Abstract

Creative thinking requires students to explore uncertainty, express tentative ideas, and take intellectual risks, yet the role of perceived school safety as a broader learning-context condition for creative thinking remains underexamined. Using data from the 2022 cycle of the Programme for International Student Assessment (PISA) from 10,239 students in Finland and 5907 students in Hong Kong (China), this study estimated two-level serial mediation models to examine the within-school associations of psychological–emotional safety, social interaction safety, and physical environmental safety with creative thinking through cooperation and curiosity. Physical environmental safety showed a positive direct association with creative thinking in both systems, and this association was stronger in Hong Kong (China). Psychological–emotional safety was positively associated with creative thinking through curiosity and through the sequential pathway from cooperation to curiosity in both systems. Social interaction safety showed no direct association with creative thinking but showed a positive sequential indirect association through cooperation and curiosity in both systems, with a stronger indirect effect in Finland. Indirect effects through cooperation alone were not significant, suggesting that cooperation may be more relevant to creative thinking when accompanied by curiosity. Curiosity was positively associated with creative thinking in both systems. The findings highlight perceived school safety as a multidimensional learning-context condition associated with creative thinking and suggest that curiosity represents a proximal process linking safety-related experiences with creative outcomes.

## 1. Introduction

Creative thinking has become increasingly important in an era of rapid technological change, as students need to generate, evaluate, and refine ideas when addressing complex and uncertain problems. Drawing on Guilford’s (1950, 1956) foundational work on creative cognition, contemporary perspectives conceptualize creative thinking as a process involving the generation, evaluation, and improvement of ideas (Dechaume et al., 2024; OECD, 2023). In educational contexts, creative thinking requires students to explore possibilities, engage with uncertainty, express tentative ideas, and revise their thinking through interaction and feedback (Hennessey & Amabile, 2010; Vincent-Lancrin et al., 2019). These processes often involve intellectual risk-taking because students may need to ask questions, propose incomplete ideas, and expose their thinking to others. Intellectual risk-taking has been associated with students’ interest, creative self-efficacy, and perceptions of supportive learning environments (Beghetto, 2009).

Research on creativity in education has largely focused on instructional strategies and classroom practices. Studies on project-based learning, creativity-supportive classrooms, and classroom task design suggest that instructional experiences can create conditions that support students’ creative thinking (Beghetto & Kaufman, 2014; Chen et al., 2022; Davies et al., 2013; Vincent-Lancrin et al., 2019). Recent research has also highlighted the relevance of socio-emotional conditions, including psychological safety and respectful interaction, for creativity-related outcomes (Castro et al., 2018; Han et al., 2022). However, existing evidence has primarily examined classroom-level conditions, while broader school environments that shape students’ willingness to explore ideas, communicate emerging thoughts, and engage in creative processes remain less understood.

School safety represents one such broader contextual condition. Creative thinking requires students to approach unfamiliar problems, tolerate uncertainty, and consider alternative possibilities. Experiences of threat, fear, or social insecurity may constrain these processes by increasing concerns about mistakes, evaluation, or interpersonal consequences. Although school safety research has extensively examined academic achievement, bullying, well-being, and mental health outcomes (Cornell et al., 2021; Marsh et al., 2023; Wang & Degol, 2016), its relationship with task-based creative thinking remains less understood. Furthermore, limited research has examined the behavioral and motivational–cognitive processes that may explain how safety-related experiences are connected with creative thinking. The present study addresses this gap by examining cooperation and curiosity as potential processes linking perceived school safety with creative thinking.

### 1.1. Perceived School Safety and Creative Thinking

Previous research conceptualizes school safety as a multidimensional construct involving physical, psychological–emotional, and social aspects (Cohen et al., 2009; Srichai et al., 2013; Thapa et al., 2013). Drawing from this multidimensional perspective, the present study examines three dimensions of perceived school safety: psychological–emotional safety, social interaction safety, and physical environmental safety. Psychological–emotional safety reflects students’ internal sense of security and psychological support (Cohen et al., 2009), whereas social interaction safety concerns experiences within peer relationships, including acceptance, exclusion, and bullying-related experiences (Olweus, 2013; Thapa et al., 2013). Physical environmental safety refers to students’ perceptions of exposure to threats, violence, or unsafe conditions (Cohen et al., 2009; Srichai et al., 2013). These dimensions represent related but distinct aspects of students’ safety experiences.

Perceived school safety may contribute to creative thinking by providing cognitive, emotional, and social conditions that support exploration and idea development. Creative thinking involves identifying problems, generating alternatives, evaluating possibilities, and refining ideas, processes that require divergent and evaluative thinking (Guilford, 1956; OECD, 2023; Runco & Acar, 2012). These processes often involve uncertainty and the possibility of failure, requiring students to engage in intellectual risk-taking and remain open to alternative possibilities (Beghetto, 2009; Hennessey & Amabile, 2010). When students experience threat or insecurity, attention and cognitive resources may be directed toward monitoring risks and avoiding negative consequences. Cognitive-load and attentional-control perspectives suggest that threat-related demands may reduce the cognitive resources available for complex thinking and problem solving by increasing attention toward potential threats (Eysenck et al., 2007; Sweller et al., 2019).

Physical environmental safety may support creative thinking by reducing threat-related cognitive demands. Unsafe environments can increase vigilance toward potential threats, limiting the cognitive resources available for complex thinking and problem solving. Research on exposure to threatening environments has shown that violence exposure is associated with poorer developmental and academic outcomes among adolescents (Henrich et al., 2004). Therefore, a physically safe learning environment may provide conditions that support the cognitive processes involved in idea generation, evaluation, and refinement.

Psychological–emotional safety may be particularly relevant to creative thinking because creativity requires students to take intellectual risks. Students often need to ask unconventional questions, propose incomplete ideas, acknowledge uncertainty, and revise their thinking after receiving feedback. Psychological safety theory suggests that individuals are more likely to speak up, learn from mistakes, and engage in exploratory behaviors when they perceive their environment as supportive (Edmondson, 1999). In educational settings, psychological safety has been associated with creativity through psychological empowerment (Han et al., 2022), and respectful listening has been shown to support idea expression by reducing concerns about negative evaluation (Castro et al., 2018).

Social interaction safety may influence creative thinking through peer communication and collaborative processes. Creative ideas often develop through interaction with others, as students exchange perspectives, receive feedback, and reconsider initial interpretations. Experiences involving bullying, exclusion, or peer rejection may discourage students from expressing unusual ideas or participating in exploratory discussions (Marsh et al., 2023; Olweus, 2013). Supportive peer relationships can provide opportunities for dialogue, perspective taking, and idea elaboration, which are important processes in collaborative creativity (Dillenbourg, 1999; Sawyer, 2007).

Taken together, these arguments suggest that perceived school safety represents an important contextual condition that may support creative thinking through cognitive, emotional, and social processes.

**H1.**  *The three dimensions of perceived school safety are positively associated with students’ creative thinking*.

### 1.2. Cooperation as a Behavioral Pathway

Cooperation represents a social–behavioral pathway through which perceived school safety may relate to creative thinking. Creative thinking often develops through interaction with others, as students encounter different perspectives, exchange ideas, and refine their initial thoughts through discussion and feedback. Cooperative experiences may therefore provide an important social context for creative thinking by encouraging students to compare interpretations, reconsider assumptions, and elaborate on emerging ideas.

From a social constructivist perspective, learning and cognition are shaped through interaction, negotiation, and the co-construction of meaning (Vygotsky, 1978). Cooperation allows students to exchange ideas, encounter alternative perspectives, receive feedback, and jointly solve problems. Collaborative learning research similarly suggests that peer interaction supports knowledge construction by enabling learners to coordinate perspectives, explain their reasoning, and respond to others’ contributions (Dillenbourg, 1999). These processes are closely connected to creative thinking because developing novel ideas often involves combining information, considering alternative viewpoints, and refining initial solutions through interaction with others (Sawyer, 2007).

Perceived school safety may facilitate cooperation by creating conditions in which students feel more comfortable interacting with peers. When students experience supportive and respectful environments, they may be more willing to participate in group activities, share tentative ideas, and engage in collaborative problem solving (Edmondson, 1999; Thapa et al., 2013). In contrast, experiences involving fear, exclusion, or peer hostility may discourage open communication and reduce students’ willingness to participate in social interactions (Olweus, 2013).

Research on adolescent development also suggests that positive interpersonal functioning is related to creative engagement, highlighting the importance of social processes in creative development (González Moreno & Molero Jurado, 2024). Although cooperation and social skills represent different constructs, both reflect social processes that influence how students engage with others during learning activities. Through these interactions, students may gain access to diverse perspectives and feedback that support creative thinking.

These arguments suggest that cooperation may serve as a behavioral pathway linking perceived school safety with creative thinking.

**H2.**  *Cooperation mediates the associations between the three dimensions of perceived school safety and creative thinking*.

### 1.3. Curiosity as a Motivational–Cognitive Pathway

Curiosity represents a motivational–cognitive pathway linking perceived school safety to creative thinking. Creative thinking requires students to explore unfamiliar possibilities, ask questions, seek alternatives, and remain engaged with problems that do not have immediate solutions. Curiosity supports these processes by motivating students to resolve uncertainty, acquire information, and examine novel possibilities.

Curiosity is commonly understood as a motivational state involving the desire to acquire information, resolve knowledge gaps, and explore uncertainty (Kidd & Hayden, 2015; Loewenstein, 1994). Research on curiosity suggests that it encourages exploration and information seeking, particularly when individuals encounter novel or complex situations (Jirout & Klahr, 2012; Kashdan et al., 2009). These exploratory tendencies are closely related to creative thinking because creative cognition involves exploration, information seeking, questioning assumptions, generating alternatives, combining information, and revising ideas (OECD, 2023).

Perceived school safety may support curiosity by influencing students’ willingness to approach unfamiliar and uncertain experiences. Exploration often involves ambiguity, mistakes, and the possibility of negative evaluation. Students who anticipate ridicule, failure, or rejection may become less willing to ask questions or pursue unfamiliar ideas (Beghetto, 2009; Edmondson, 1999). In contrast, supportive environments may encourage students to engage with novelty and complexity by reducing concerns about interpersonal risks.

The appraisal theory of interest suggests that curiosity and interest are more likely to emerge when individuals perceive a situation as novel or complex while also believing that they can understand or manage the challenge (Silvia, 2005). Thus, perceived safety may provide students with the psychological conditions needed to approach uncertainty as an opportunity for exploration.

These arguments suggest that curiosity may serve as a motivational pathway linking perceived school safety with creative thinking.

**H3.**  *Curiosity mediates the associations between the three dimensions of perceived school safety and creative thinking*.

### 1.4. The Sequential Pathway from Cooperation to Curiosity

Cooperation may also contribute to curiosity through students’ social learning experiences. While cooperation provides opportunities for students to exchange ideas and encounter different perspectives, curiosity reflects the motivation to explore questions, resolve uncertainty, and seek new information. Cooperative interactions may therefore create conditions that stimulate curiosity by exposing students to unfamiliar ideas, alternative explanations, and unresolved problems.

In cooperative learning contexts, students are exposed to peers’ different ideas, strategies, and interpretations. These interactions can increase perceived novelty and complexity, which are important antecedents of interest and curiosity (Silvia, 2005). From an information-gap perspective, encounters with new perspectives or incomplete understanding can generate awareness of knowledge gaps and motivate further exploration (Loewenstein, 1994). Peer discussion and collaborative inquiry may therefore stimulate curiosity by introducing students to questions and possibilities that extend beyond their existing knowledge (Jirout & Klahr, 2012).

Cooperation also provides students with social and cognitive resources, including peer support, feedback, and shared problem-solving strategies (Dillenbourg, 1999). Such interactions may increase students’ confidence when approaching unfamiliar problems and encourage exploratory engagement. Collaborative learning environments can support creative processes by allowing students to negotiate meanings, combine perspectives, and develop ideas through interaction with others (Sawyer, 2007).

Together, these arguments suggest a sequential pathway in which perceived school safety supports cooperative interaction, cooperative interaction exposes students to diverse perspectives and unresolved questions, and these experiences stimulate curiosity that contributes to creative thinking. A safe school environment may facilitate open interaction by reducing concerns about negative evaluation and interpersonal risk. Through cooperative exchanges, students may encounter diverse perspectives and receive feedback that stimulates further exploration. Curiosity may then support the generative and evaluative processes involved in creative thinking.

**H4.**  *Cooperation and curiosity sequentially mediate the associations between the three dimensions of perceived school safety and creative thinking*.

### 1.5. The Present Study

Despite growing evidence on creativity-supportive instruction and classroom environments, less is known about how broader school conditions and underlying psychosocial processes are related to students’ creative thinking. The present study examines perceived school safety as a multidimensional contextual condition and investigates whether its relationship with creative thinking operates through cooperation, curiosity, and the sequential pathway from cooperation to curiosity. Specifically, it examines how different safety experiences are associated with creative thinking through behavioral and motivational–cognitive processes.

Using PISA 2022 data from Finland and Hong Kong (China), this study examines the proposed psychosocial pathways across two educational contexts. The PISA 2022 framework provides a unique opportunity for this investigation by combining internationally comparable, performance-based assessment of creative thinking with contextual information on students’ learning environments (OECD, 2023, 2024a). These two settings provide meaningful contextual variation for examining whether the relationships among perceived school safety, cooperation, curiosity, and creative thinking show consistent or context-sensitive patterns across educational settings, thereby extending research on creative thinking beyond instructional conditions and clarifying the behavioral and motivational–cognitive pathways linking school environments with creative outcomes.

## 2. Materials and Methods

### 2.1. Data Source and Study Design

This study used publicly available data from PISA 2022, conducted by the Organisation for Economic Co-operation and Development (OECD). PISA assesses students aged between 15 years 3 months and 16 years 2 months at the beginning of the assessment period, typically near the end of compulsory education. It collects internationally comparable information on student performance, family background, school experiences, and learning environments through standardized cognitive assessments and background questionnaires (OECD, 2023, 2024b). PISA 2022 employed a two-stage stratified sampling design, in which schools were sampled first and eligible students were then sampled within participating schools (OECD, 2024b). In the 2022 cycle, creative thinking was assessed as the innovative domain, focusing on students’ capacity to generate diverse ideas, generate creative ideas, and evaluate and improve ideas (OECD, 2023, 2024a).

The present study adopted a cross-sectional comparative design and focused on Finland and Hong Kong (China), both of which participated in the PISA 2022 creative thinking assessment. These two education systems were selected because they provide theoretically informative contexts for examining the robustness of the proposed psychosocial pathways linking school safety to creative thinking. Finland and Hong Kong (China) differ in several educational characteristics, including instructional traditions, learning environments, and patterns of educational organization. Finnish education has been characterized by an emphasis on student autonomy, teacher professionalism, collaborative learning, and holistic development (Sahlberg, 2011), whereas Hong Kong’s education system has traditionally placed greater emphasis on academic achievement, structured learning processes, and examination performance within a highly competitive educational environment (Morris, 1996). These contextual differences provide an opportunity to examine whether the associations among perceived school safety, cooperation, curiosity, and creative thinking demonstrate consistent patterns across distinct educational settings. The comparison therefore focuses on the contextual robustness of the proposed model while avoiding interpretations of system-level differences beyond the scope of the present data. In addition, both systems administered the creative thinking assessment data and the relevant student background questionnaire modules, with student- and school-level data available for analysis.

The study sample comprised 10,239 students from 241 schools in Finland and 5907 students from 163 schools in Hong Kong (China). In Finland, 51.2% of the participating students were male; in Hong Kong (China), 52.1% were male.

### 2.2. Measures

#### 2.2.1. Creative Thinking

The dependent variable in this study was students’ creative thinking performance. In PISA 2022, creative thinking was assessed through cognitive test tasks rather than through student self-reports. The PISA framework defines creative thinking as students’ capacity to engage productively in the generation, evaluation, and improvement of ideas that may lead to original and effective solutions, advances in knowledge, or impactful expressions of imagination (OECD, 2023, 2024a). For measurement purposes, the PISA 2022 creative thinking assessment was organized around three cognitive facets: generating diverse ideas, generating creative ideas, and evaluating and improving ideas. These facets capture both divergent processes, such as producing varied and original ideas, and convergent processes, such as evaluating and refining ideas (OECD, 2023).

Because PISA uses item response theory and a matrix-sampling design, students’ creative thinking performance is reported through multiple plausible values rather than a single observed test score. In this study, creative thinking was represented by the ten PISA 2022 creative thinking plausible values: PV1CRTH_NC to PV10CRTH_NC.

#### 2.2.2. Perceived School Safety

The focal independent variable was students’ perceived school safety. In line with the theoretical framework of this study, school safety was operationalized in three dimensions: psychological–emotional safety, social interaction safety, and physical environmental safety. All three indicators were derived from the PISA 2022 student questionnaire. Therefore, they should be interpreted as students’ subjective perceptions of safety and self-reported exposure to safety-related risks, rather than as direct measures of schools’ objective safety infrastructure, security policies, or administrative practices. Conceptually, these measures are located within the broader domain of school climate, in which safety, order, interpersonal relations, and perceived support are central components of students’ learning environment (OECD, 2023; Wang & Degol, 2016).

Psychological–emotional safety was measured using the PISA 2022 index of students’ perceived safety at school (FEELSAFE). This index captures students’ subjective feelings of safety on their way to school, on their way home from school, in classrooms, and in other school spaces such as cafeterias and restrooms. The underlying item set consists of four four-point Likert-type items. Higher scores indicate a higher level of perceived psychological–emotional safety. In the present sample, the internal consistency of the underlying item set was high, with Cronbach’s α = 0.917 in Finland and α = 0.928 in Hong Kong (China).

Social interaction safety was derived from the PISA 2022 index of bullying victimization (BULLIED). The original BULLIED index reflects the frequency with which students reported experiencing peer exclusion, teasing, threats, property damage, physical aggression, and malicious rumors during the previous 12 months. The underlying item set consists of nine four-point Likert-type items, such as “Other students left me out of things on purpose.” To align the direction of this measure with the concept of safety, the BULLIED index was multiplied by −1. Thus, higher scores on the derived social interaction safety variable indicate lower reported exposure to bullying victimization and, therefore, a higher level of perceived social interaction safety. In the present sample, the internal consistency of the underlying item set was Cronbach’s α = 0.833 in Finland and α = 0.821 in Hong Kong (China).

Physical environmental safety was derived from the PISA 2022 index of school safety risks (SCHRISK). The original SCHRISK index reflects students’ reports of safety-related incidents at school during the previous four weeks, including vandalism, fighting, gangs, threats of harm, and weapons at school. The underlying item set consists of five dichotomous items. To ensure a consistent interpretive direction across the school safety variables, the SCHRISK index was multiplied by −1. Thus, higher scores on the derived physical environmental safety variable indicate lower reported exposure to school safety risks and, therefore, a higher level of perceived physical environmental safety. In the present sample, the internal consistency of the underlying item set was Cronbach’s α = 0.764 in Finland and α = 0.745 in Hong Kong (China).

#### 2.2.3. Cooperation

Cooperation was included as the first mediator in this study. It was measured using the PISA 2022 index of students’ cooperation orientation (COOPAGR). This index captures students’ self-reported cooperative attitudes and behaviors in interpersonal and school-related contexts. The underlying item set consists of ten five-point Likert-type items, ranging from 1 = strongly disagree to 5 = strongly agree. Example items include “I like helping people,” “I cooperate well with other people,” “I like to cooperate with my classmates,” and “When I am part of a team, I work better.” Some negatively worded items, such as “I argue with other people” and “I avoid cooperating with other students,” were reverse-coded in the construction of the index. Higher scores indicate a higher level of cooperation. In the present sample, the internal consistency of the underlying item set was Cronbach’s α = 0.817 in Finland and α = 0.854 in Hong Kong (China).

#### 2.2.4. Curiosity

Curiosity was included as the second mediator in this study. It was measured using the PISA 2022 index of students’ curiosity (CURIOAGR). This index reflects students’ interest in new ideas, tendency to ask questions, and willingness to explore unfamiliar or uncertain topics. The underlying item set consists of ten five-point Likert-type items, ranging from 1 = strongly disagree to 5 = strongly agree. Example items include “I am curious about many different things,” “I like asking questions,” “I want to know how things work,” “I like learning new things at school,” and “I spend time finding out more about things that interest me.” Some negatively worded items, such as “I find learning new things boring,” were reverse-coded in the construction of the index. Higher scores indicate a higher level of curiosity. In the present sample, the internal consistency of the underlying item set was Cronbach’s α = 0.834 in Finland and α = 0.819 in Hong Kong (China).

#### 2.2.5. Covariates

Gender and family socioeconomic status were included as theoretically relevant covariates in all models. Gender was coded as a dichotomous covariate, with female students coded as 1 and male students coded as 2. Family socioeconomic status was measured using the PISA index of economic, social, and cultural status (ESCS). ESCS is a composite index constructed by the OECD based on information about parents’ education, parents’ occupational status, and home possessions. Higher ESCS values indicate a higher level of family socioeconomic and cultural resources. Gender and ESCS were included because previous research has documented their associations with students’ creative thinking and educational experiences. Specifically, gender differences have been reported in creative thinking performance (He & Wong, 2021; Taylor, 2025), and ESCS has been positively associated with adolescents’ creative outcomes by reflecting differences in family resources and developmental opportunities (Kuspanova et al., 2025; Parsasirat et al., 2013). Variables representing students’ psychological experiences and learning processes, such as cooperation and curiosity, were incorporated into the proposed mediation framework because they were theoretically positioned as explanatory processes linking perceived school safety to creative thinking.

### 2.3. Statistical Analysis

Given the hierarchical structure of the PISA 2022 data, with students nested within schools, this study employed two-level multilevel models rather than single-level regression models. Multilevel modeling is appropriate for clustered educational data because it accounts for the non-independence of observations within the same school, separates within-school from between-school sources of variation, and produces more appropriate standard errors when the independence assumption of ordinary regression is violated (Raudenbush & Bryk, 2002). In the context of mediation analysis with nested data, a multilevel structural equation modeling framework also helps avoid conflating within-school and between-school components of mediation effects (Preacher et al., 2010).

All analyses were conducted in Mplus 8.3. The final student weight, W_FSTUWT, was applied to adjust for unequal selection probabilities and nonresponse in the PISA sampling design. The substantive mediation model was specified at the student level. Student-level variables involved in the mediation paths were group-mean centered within schools so that the estimated path coefficients represented within-school associations. At the school level, the model included random intercepts only; no school-level predictors or random slopes were specified.

The proportion of missing data for the analytic variables ranged from 0% to 5%. Missing data were handled using full-information maximum likelihood (FIML), which estimates model parameters based on all available information under the assumption that data are missing at random (Schafer & Graham, 2002). Model parameters were then estimated using the robust maximum likelihood estimator (MLR), which provides standard errors and test statistics that are robust to non-normality (Muthén & Muthén, 1998–2017). Because standard nonparametric bootstrap confidence intervals are not available for two-level models in Mplus, confidence intervals were obtained using the default symmetric confidence intervals based on the model-estimated standard errors.

Because PISA reports student achievement through plausible values rather than a single observed test score, the ten plausible values for creative thinking were analyzed separately. Specifically, the same two-level serial mediation model was estimated ten times, with each of the ten creative thinking plausible values used as the outcome variable in turn. Parameter estimates and standard errors were then pooled according to Rubin’s rules (Rubin, 1987). The final point estimate was calculated as the average of the ten estimates, and the total variance incorporated both the average within-PV variance and the between-PV variance. This procedure accounts for measurement uncertainty in PISA achievement estimates and is consistent with recommended practice for analyzing large-scale assessment data (Kaplan & Su, 2016; OECD, 2024b). To examine whether the estimated pathways differed between Finland and Hong Kong (China), Wald tests were conducted to compare the corresponding path coefficients and indirect effects across the two education systems (Wald, 1943).

## 3. Results

### 3.1. Descriptive Statistics and Correlations

The weighted descriptive statistics and correlations for the main study variables are presented in Table 1 for Finland and Hong Kong (China).

### 3.2. Intraclass Correlations

Prior to estimating the multilevel mediation models, unconditional two-level models were examined to assess the extent of between-school variation in creative thinking. Intraclass correlation coefficients (ICC) were calculated separately for the ten creative thinking plausible values and then averaged across plausible values. ICCs indicated that 10.7% of the variance in creative thinking in Finland and 27.7% in Hong Kong (China) was attributable to differences between schools. These results suggest that creative thinking varied meaningfully across schools, particularly in Hong Kong (China), thereby warranting the use of two-level models to account for the nested structure of students within schools.

### 3.3. Multilevel Serial Mediation Model Results

**Figure 1:**
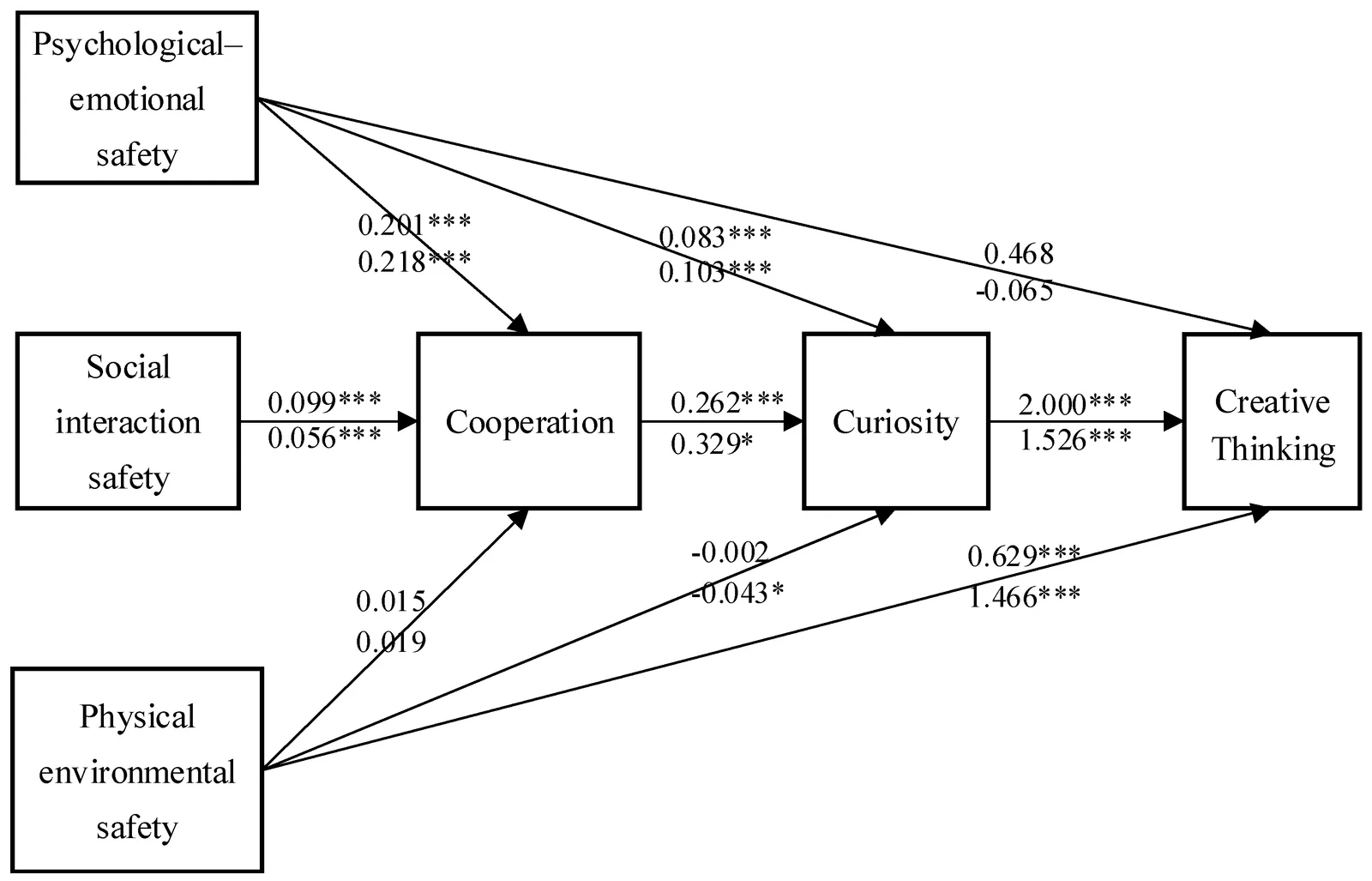
Within-School Serial Mediation Pathways for Finland and Hong Kong (China). *Note*. Values on the paths are unstandardized regression coefficients (B); the first line represents Finland and the second line represents Hong Kong (China). All models controlled for gender and ESCS, but these paths are omitted for visual clarity. * *p* < 0.05, *** *p* < 0.001.

Table 2 reports the direct and component paths from the two-level serial mediation models. The models included direct paths from the three dimensions of perceived school safety to creative thinking, as well as indirect paths through cooperation, curiosity, and the sequential path from cooperation to curiosity. Figure 1 provides a visual summary of the within-school pathways for Finland and Hong Kong (China). Gender and ESCS were controlled in all models.

The component paths showed that psychological–emotional safety was positively associated with both cooperation and curiosity in Finland and Hong Kong (China). Social interaction safety was also positively associated with cooperation in both education systems. In contrast, physical environmental safety was not significantly associated with cooperation in either system. The path from cooperation to curiosity was positive and statistically significant in both Finland and Hong Kong (China), indicating that students with higher levels of cooperation also tended to report higher levels of curiosity within the same school.

For the paths to creative thinking in the full mediation model, physical environmental safety was positively associated with creative thinking in both Finland and Hong Kong (China). The direct association between psychological–emotional safety and creative thinking in Finland approached the conventional threshold of statistical significance (*B* = 0.47, *SE* = 0.238, *p* = 0.050, 95% CI [0.000, 0.935]), whereas this path was not significant in Hong Kong (China). Social interaction safety was not directly associated with creative thinking in either system. Cooperation was not significantly associated with creative thinking in either system after controlling for the other variables in the model. By contrast, curiosity was positively associated with creative thinking in both Finland and Hong Kong (China). These results suggest that curiosity was the more proximal predictor of creative thinking, whereas cooperation was linked to creative thinking mainly through its association with curiosity.

### 3.4. Indirect Effects

Table 3 reports the indirect effects through cooperation, through curiosity, and through the sequential pathway from cooperation to curiosity.

None of the indirect effects through cooperation alone was statistically significant in either education system. Thus, the hypothesized single-mediator role of cooperation was not supported.

The indirect effects through curiosity showed a more differentiated pattern. In both education systems, psychological–emotional safety was positively associated with creative thinking through curiosity. The within-system significance pattern for the other two safety dimensions was not identical across the two systems. In Finland, social interaction safety showed a significant negative indirect association with creative thinking through curiosity, whereas this indirect effect was not significant in Hong Kong (China). Conversely, physical environmental safety showed a significant negative indirect association with creative thinking through curiosity in Hong Kong (China) but not in Finland.

The sequential indirect effects through cooperation and curiosity were significant for psychological–emotional safety and social interaction safety in both education systems. Psychological–emotional safety was positively associated with creative thinking through the sequence of cooperation and curiosity in both Finland and Hong Kong (China). Social interaction safety also showed a significant positive sequential indirect association in both systems. By contrast, the sequential indirect effect of physical environmental safety was not significant in either system.

Taken together, the indirect-effect results indicate that cooperation did not function as an independent mediator between perceived school safety and creative thinking. However, cooperation formed part of a significant sequential pathway when followed by curiosity, particularly for psychological–emotional safety and social interaction safety. In this model, cooperation was more closely involved in the pathway to curiosity than in a direct association with creative thinking.

### 3.5. Cross-System Comparisons

Wald tests were conducted to compare selected direct and indirect paths between Finland and Hong Kong (China). These comparisons focused on theoretically central pathways and pathways that were statistically significant in at least one education system. The difference coefficient was computed as the Finland estimate minus the Hong Kong (China) estimate. Results are presented in Table 4.

The direct path from physical environmental safety to creative thinking differed significantly between the two education systems (Difference = −0.84, *SE* = 0.288, Wald *χ*^2^ = 8.28, *p* = 0.004), indicating that the positive direct association was stronger in Hong Kong (China) than in Finland. For the indirect pathways through curiosity, no statistically significant cross-system differences were detected. No statistically significant difference was detected for the indirect effect of psychological–emotional safety through curiosity, social interaction safety through curiosity, or physical environmental safety through curiosity.

For the sequential indirect pathways, no statistically significant difference was detected for the pathway from psychological–emotional safety to creative thinking through cooperation and curiosity. However, the sequential indirect pathway from social interaction safety to creative thinking through cooperation and curiosity was significantly stronger in Finland than in Hong Kong (China) (Difference = 0.02, *SE* = 0.011, Wald *χ*^2^ = 4.65, *p* = 0.031).

Overall, the Wald tests indicated that most theoretically central pathways did not show statistically detectable differences between Finland and Hong Kong (China). The main cross-system differences were found for the direct path from physical environmental safety to creative thinking, which was stronger in Hong Kong (China), and for the sequential indirect path from social interaction safety through cooperation and curiosity, which was stronger in Finland. Non-significant Wald tests should be interpreted only as indicating no statistically detectable difference, not as evidence of equivalence.

### 3.6. Summary of Hypothesis Testing

In summary, H1 was partially supported: physical environmental safety was positively associated with creative thinking in both Finland and Hong Kong (China), whereas the direct paths from psychological–emotional safety and social interaction safety to creative thinking were not consistently significant. H2 was not supported, as none of the indirect effects through cooperation alone was statistically significant. H3 was partially supported, with the most consistent indirect pathway observed from psychological–emotional safety to creative thinking through curiosity. H4 was partially supported, as the sequential indirect pathways through cooperation and curiosity were significant for psychological–emotional safety and social interaction safety but not for physical environmental safety.

## 4. Discussion

This study examined whether students’ perceived school safety was associated with creative thinking through cooperation and curiosity in Finland and Hong Kong (China). The findings provide a differentiated account of this relationship by showing that different safety experiences are associated with creative thinking through distinct psychosocial processes. Physical environmental safety was the most directly related to creative thinking, whereas psychological–emotional and social interaction safety were more closely related to curiosity-related and cooperation–curiosity pathways. The findings also clarify the role of cooperation by suggesting that its relevance for creative thinking depends partly on whether cooperative experiences stimulate curiosity and further exploration.

### 4.1. Perceived School Safety as a Differentiated Condition for Creative Thinking

The findings extend creativity research by highlighting perceived school safety as a broader ecological condition associated with creative thinking beyond instructional and classroom-level factors. This perspective is consistent with ecological approaches to development, which emphasize that students’ cognitive and motivational processes are shaped by the environments in which learning occurs (Bronfenbrenner, 1979). Prior research has emphasized classroom-based and pedagogical supports for creativity, including project-based learning, creativity-supportive classrooms, task design, and instructional practices (Beghetto & Kaufman, 2014; Chen et al., 2022; Davies et al., 2013; Vincent-Lancrin et al., 2019). The present results suggest that these instructional conditions operate within a broader learning environment in which students need to feel sufficiently safe to explore ideas, ask questions, and take intellectual risks.

Physical environmental safety showed the clearest direct association with creative thinking. Students who reported lower exposure to school safety risks tended to show higher creative thinking performance within the same school. This pattern is consistent with cognitive-load and attentional-control perspectives, which may help explain why students’ perceived exposure to safety risks was associated with creative thinking performance (Eysenck et al., 2007; Sweller et al., 2019). However, this interpretation concerns students’ reported exposure to safety-related risks rather than objective school infrastructure or administrative safety conditions.

The stronger association between physical environmental safety and creative thinking in Hong Kong (China) than in Finland requires cautious interpretation. Because the models estimated within-school associations and did not include school-level safety climate predictors or random slopes, this difference should not be interpreted as evidence that one education system provides a more effective safety environment for creativity. One possible explanation is that students may differ in how they experience or interpret physical safety risks across educational contexts. Such differences may reflect variations in students’ perceptions of risk and the broader conditions in which safety experiences are formed.

Psychological–emotional safety and social interaction safety did not show consistent direct associations with creative thinking. Their relevance may become more apparent through motivational and social processes. This interpretation aligns with school climate research, which considers safety, relationships, support, and belonging as related dimensions of students’ learning environments (Cohen et al., 2009; Thapa et al., 2013; Wang & Degol, 2016). It also extends school safety research, which has often examined outcomes such as achievement, bullying, well-being, and mental health (Cornell et al., 2021; Marsh et al., 2023), by showing that safety-related experiences may also be associated with higher-order cognitive outcomes such as creative thinking.

### 4.2. Curiosity as a Proximal Motivational–Cognitive Pathway

Curiosity was the most consistent mediator in the model. It was positively associated with creative thinking in both educational contexts, and psychological–emotional safety showed a consistent positive indirect association with creative thinking through curiosity. This finding highlights the importance of students’ willingness to explore uncertainty when considering how school experiences relate to creative thinking.

Curiosity involves the desire to acquire information, resolve knowledge gaps, and explore uncertainty (Kidd & Hayden, 2015; Loewenstein, 1994). These characteristics closely align with creative thinking, which requires students to search for alternatives, consider unfamiliar possibilities, and revise initial ideas. Students who experience emotionally supportive school environments may be more willing to ask questions, approach novelty, and persist when solutions are not immediately available.

This result also clarifies how psychological–emotional safety may matter for creative thinking. Psychological safety theory argues that individuals are more likely to express ideas, learn from mistakes, and take interpersonal risks when they perceive their environment as supportive (Edmondson, 1999). Previous educational research has linked psychological safety with creativity through psychological empowerment, and respectful listening has been shown to facilitate idea expression by reducing concerns about negative evaluation (Castro et al., 2018; Han et al., 2022). The present findings extend this literature by suggesting that psychological–emotional safety may support curiosity-related exploration, which is more directly connected with creative thinking.

The unexpected negative indirect effects through curiosity observed for some safety dimensions should be interpreted cautiously. In Finland, social interaction safety showed a negative indirect association through curiosity, whereas in Hong Kong (China), physical environmental safety showed a similar pattern. These effects may reflect the statistical complexity of modeling related dimensions of perceived safety simultaneously rather than indicating that lower safety promotes curiosity or creative thinking. The non-significant Wald tests further suggest that these within-system patterns should not be interpreted as robust differences between Finland and Hong Kong (China).

### 4.3. Cooperation Within a Sequential Curiosity-Related Pathway

The role of cooperation in the association between perceived school safety and creative thinking was more complex than a direct mediation model would suggest. Cooperation alone did not show a significant indirect association with creative thinking, whereas the sequential pathway through cooperation and curiosity was significant for psychological–emotional safety and social interaction safety. These findings suggest that cooperative experiences may become relevant to creative thinking when they stimulate curiosity and further exploration.

This pattern may help explain why cooperation did not directly translate into stronger creative thinking performance. Cooperation provides opportunities for students to encounter different perspectives, exchange ideas, and receive feedback, which can create cognitive stimulation and encourage further exploration (Dillenbourg, 1999; Sawyer, 2007). From a social constructivist perspective, learning develops through interaction, negotiation, and the co-construction of meaning (Vygotsky, 1978). However, exposure to different ideas alone may not be sufficient for creative thinking. Students also need to engage with uncertainty, ask questions, and examine alternative possibilities, processes that are closely related to curiosity.

The present findings extend existing understandings of cooperation in creativity development by suggesting that cooperation may function as a social condition that supports creative thinking through motivational processes. Previous research has emphasized the importance of collaborative interaction for idea development and knowledge construction (Dillenbourg, 1999; Sawyer, 2007). The current findings add a more specific explanation by showing that cooperative experiences may be more closely connected with creative thinking when they activate curiosity-related exploration. In this sense, cooperation may provide the social context in which curiosity develops, while curiosity represents a more proximal process linking social experiences with creative thinking.

### 4.4. Contextual Robustness and Boundary Conditions Across Educational Settings

The cross-system comparison suggested substantial consistency in the proposed psychosocial pathways while also indicating that the strength of some associations may vary across educational contexts. This pattern suggests that the relationship between school safety and creative thinking may involve processes that are partly robust across settings but remain sensitive to contextual conditions.

Previous research on creativity has highlighted the importance of supportive learning environments, curiosity, and exploratory processes in creative thinking development (Beghetto & Kaufman, 2014; Vincent-Lancrin et al., 2019). Similarly, school climate research has emphasized that students’ experiences of safety, relationships, and support are shaped by the broader educational environments in which learning occurs (Cohen et al., 2009; Wang & Degol, 2016). Together, these findings suggest that safety-related psychosocial processes show some robustness across the two educational contexts examined, while specific pathways remain sensitive to contextual conditions.

The observed differences between Finland and Hong Kong (China) should be interpreted cautiously. Although the two settings provide informative contexts for examining the proposed model, the present study does not directly measure classroom practices, institutional characteristics, or cultural mechanisms that may account for contextual variation. Future research should examine how these contextual factors shape the relationship between school safety experiences and creative thinking across a broader range of educational systems.

### 4.5. Theoretical and Practical Implications

For educational practice, fostering creative thinking requires attention to the environments in which students generate and develop ideas. Creating conditions in which students feel safe to ask questions, share tentative ideas, and explore uncertainty may complement instructional approaches designed to support creativity. The present study extends this perspective by showing that students’ safety-related experiences may also shape the conditions under which creative thinking occurs.

The findings further clarify the role of cooperation in creativity development. Cooperative experiences may support creative thinking when they encourage curiosity, exploration, and engagement with alternative ideas. This suggests that educational practices should pay attention to the quality of peer interaction rather than cooperation as an isolated activity. Learning environments that allow students to ask questions, express tentative ideas, and explore uncertainty may provide more supportive conditions for creative thinking.

Similar patterns observed across Finland and Hong Kong (China) suggest that the relationship among school safety, cooperation, curiosity, and creative thinking may have relevance across different educational contexts. At the same time, differences in specific pathways highlight the importance of considering contextual conditions when interpreting how learning environments relate to creative development.

### 4.6. Limitations and Future Research

Several limitations should be considered. First, the cross-sectional nature of PISA 2022 data limits causal interpretation of the proposed pathways. Although the model specified associations among perceived school safety, cooperation, curiosity, and creative thinking, the data cannot establish temporal ordering or causal direction. Future longitudinal and intervention studies are needed to examine these relationships over time.

Second, the measures of perceived school safety, cooperation, and curiosity were based on student-reported PISA indicators. These measures capture important aspects of students’ experiences but may not fully represent the complexity of school environments or the psychological processes underlying creative thinking. Future research could combine task-based creative thinking assessments with more targeted measures of safety experiences, motivational processes, and interpersonal interactions.

Third, the analyses focused on student-level within-school associations and did not examine how school-level conditions, such as safety climate and classroom interaction patterns, may shape these relationships. Future multilevel research could examine how different levels of learning environments jointly influence creative thinking development.

Finally, the comparison between Finland and Hong Kong (China) provides informative contexts for examining the proposed model but cannot represent all educational systems. The observed differences should be interpreted as contextual variations, with specific cultural mechanisms requiring further investigation. Future research across a broader range of educational settings is needed to further examine the boundary conditions of these pathways.

## 5. Conclusions

The findings suggest that perceived school safety represents an important contextual condition for creative thinking development, extending attention beyond instructional practices to the broader environments in which students learn. Safety-related experiences were linked with creative thinking through distinct psychosocial processes, with curiosity playing a particularly important role in connecting supportive learning environments with creative exploration. The findings further suggest that cooperation may contribute to creative thinking when it stimulates curiosity and engagement with unfamiliar ideas. The broadly consistent patterns observed across Finland and Hong Kong (China) suggest that safety-related psychosocial processes may extend beyond the specific settings examined, although their manifestations may depend on contextual conditions. Given the cross-sectional nature of PISA data, these findings should be interpreted as associations and encourage further research on how learning environments shape students’ creative thinking development.

## Figures and Tables

**Table 1:** Descriptive Statistics and Correlations for the Main Study Variables in Finland and Hong Kong (China).

Education System	Variable	*M*	*SD*	1	2	3	4	5
Finland	1. Psychological–emotional safety	0.00	0.932	—				
	2. Social interaction safety	0.00	0.983	0.23 ***	—			
	3. Physical environmental safety	0.00	1.019	0.11 ***	0.33 ***	—		
	4. Curiosity	0.00	0.912	0.14 ***	0.01	0.01	—	
	5. Cooperation	0.00	0.979	0.20 ***	0.14 ***	0.06 ***	0.30 ***	—
	6. Creative thinking	35.50	11.148	0.06 ***	0.02 *	0.05 ***	0.24 ***	0.10 ***
Hong Kong (China)	1. Psychological–emotional safety	0.00	0.950	—				
	2. Social interaction safety	0.00	1.003	0.10 ***	—			
	3. Physical environmental safety	0.00	0.779	0.09 ***	0.32 ***	—		
	4. Curiosity	0.00	0.919	0.19 ***	−0.01	−0.02	—	
	5. Cooperation	0.00	0.837	0.26 ***	0.10 ***	0.07 ***	0.33 ***	—
	6. Creative thinking	31.85	9.437	0.03 *	0.11 ***	0.16 ***	0.17 ***	0.08 ***

*Note*. * *p* < 0.05, *** *p* < 0.001. Creative thinking was described using the arithmetic mean of the ten PISA 2022 creative thinking plausible values (PV1CRTH_NC–PV10CRTH_NC). This averaged score was used only for descriptive statistics and correlations. Descriptive statistics and correlations are weighted.

**Table 2:** Direct and Component Paths in the Multilevel Serial Mediation Models.

Education System	Path	*B*	*SE*	*p*	95% CI
Finland	Psychological–emotional safety → Cooperation	0.201	0.013	<0.001	[0.177, 0.226]
	Social interaction safety → Cooperation	0.099	0.011	<0.001	[0.077, 0.121]
	Physical environmental safety → Cooperation	0.015	0.011	0.177	[−0.007, 0.037]
	Psychological–emotional safety → Curiosity	0.083	0.011	<0.001	[0.060, 0.105]
	Social interaction safety → Curiosity	−0.045	0.011	<0.001	[−0.068, −0.023]
	Physical environmental safety → Curiosity	−0.002	0.011	0.876	[−0.024, 0.020]
	Cooperation → Curiosity	0.262	0.014	<0.001	[0.234, 0.290]
	Psychological–emotional safety → Creative thinking	0.468	0.238	0.050	[0.000, 0.935]
	Social interaction safety → Creative thinking	−0.035	0.170	0.839	[−0.367, 0.298]
	Physical environmental safety → Creative thinking	0.629	0.167	<0.001	[0.301, 0.956]
	Cooperation → Creative thinking	0.015	0.190	0.938	[−0.357, 0.387]
	Curiosity → Creative thinking	2.000	0.236	<0.001	[1.538, 2.463]
Hong Kong (China)	Psychological–emotional safety → Cooperation	0.218	0.013	<0.001	[0.192, 0.240]
	Social interaction safety → Cooperation	0.056	0.011	<0.001	[0.035, 0.074]
	Physical environmental safety → Cooperation	0.019	0.016	0.226	[−0.012, 0.045]
	Psychological–emotional safety → Curiosity	0.103	0.013	<0.001	[0.076, 0.125]
	Social interaction safety → Curiosity	−0.022	0.014	0.102	[−0.049, 0.000]
	Physical environmental safety → Curiosity	−0.043	0.020	0.033	[−0.083, −0.010]
	Cooperation → Curiosity	0.329	0.025	<0.001	[0.280, 0.369]
	Psychological–emotional safety → Creative thinking	−0.065	0.206	0.750	[−0.469, 0.338]
	Social interaction safety → Creative thinking	0.289	0.198	0.145	[−0.099, 0.677]
	Physical environmental safety → Creative thinking	1.466	0.237	<0.001	[1.002, 1.930]
	Cooperation → Creative thinking	0.048	0.263	0.856	[−0.468, 0.563]
	Curiosity → Creative thinking	1.526	0.222	<0.001	[1.092, 1.961]

*Note*. Estimates are unstandardized coefficients. Gender and ESCS were controlled in all models.

**Table 3:** Indirect Effects in the Multilevel Serial Mediation Models.

Education System	Indirect Pathway	*B*	*SE*	*p*	95% CI
Finland	Psychological–emotional safety → Cooperation → Creative thinking	0.003	0.038	0.938	[−0.072, 0.078]
	Social interaction safety → Cooperation → Creative thinking	0.001	0.019	0.937	[−0.035, 0.038]
	Physical environmental safety → Cooperation → Creative thinking	0.001	0.003	0.942	[−0.006, 0.006]
	Psychological–emotional safety → Curiosity → Creative thinking	0.166	0.029	<0.001	[0.110, 0.222]
	Social interaction safety → Curiosity → Creative thinking	−0.091	0.024	<0.001	[−0.139, −0.043]
	Physical environmental safety → Curiosity → Creative thinking	−0.003	0.022	0.878	[−0.047, 0.041]
	Psychological–emotional safety → Cooperation → Curiosity → Creative thinking	0.105	0.015	<0.001	[0.077, 0.134]
	Social interaction safety → Cooperation → Curiosity → Creative thinking	0.052	0.009	<0.001	[0.034, 0.069]
	Physical environmental safety → Cooperation → Curiosity → Creative thinking	0.008	0.006	0.193	[−0.004, 0.020]
Hong Kong (China)	Psychological–emotional safety → Cooperation → Creative thinking	0.010	0.057	0.857	[−0.102, 0.123]
	Social interaction safety → Cooperation → Creative thinking	0.003	0.015	0.857	[−0.027, 0.032]
	Physical environmental safety → Cooperation → Creative thinking	0.001	0.006	0.873	[−0.011, 0.013]
	Psychological–emotional safety → Curiosity → Creative thinking	0.157	0.029	<0.001	[0.099, 0.214]
	Social interaction safety → Curiosity → Creative thinking	−0.034	0.021	0.109	[−0.075, 0.008]
	Physical environmental safety → Curiosity → Creative thinking	−0.066	0.032	0.042	[−0.129, −0.002]
	Psychological–emotional safety → Cooperation → Curiosity → Creative thinking	0.109	0.019	<0.001	[0.072, 0.147]
	Social interaction safety → Cooperation → Curiosity → Creative thinking	0.028	0.007	<0.001	[0.015, 0.041]
	Physical environmental safety → Cooperation → Curiosity → Creative thinking	0.010	0.008	0.245	[−0.007, 0.026]

*Note*. Estimates are unstandardized indirect effects. Gender and ESCS were controlled in all models.

**Table 4:** Wald Tests for Cross-System Differences in Theoretical Paths.

Path	Difference	*SE*	Wald *χ*^2^	*p*
Physical environmental safety → Creative thinking	−0.84	0.288	8.28	0.004
Psychological–emotional safety → Curiosity → Creative thinking	0.01	0.040	0.05	0.824
Social interaction safety → Curiosity → Creative thinking	−0.05	0.032	3.15	0.076
Physical environmental safety → Curiosity → Creative thinking	0.06	0.039	2.51	0.113
Psychological–emotional safety → Cooperation → Curiosity → Creative thinking	−0.01	0.024	0.03	0.862
Social interaction safety → Cooperation → Curiosity → Creative thinking	0.02	0.011	4.65	0.031

*Note*. Difference = Finland estimate—Hong Kong (China) estimate. Estimates are unstandardized difference coefficients.

## Data Availability

The PISA 2022 student datasets analysed during the current study are publicly available from the OECD PISA database at https://www.oecd.org/pisa/data/2022database/ (accessed on 25 May 2025). No new datasets were generated by the authors.
